# A Word of Caution to Woolen Mill Operatives

**Published:** 1886-09

**Authors:** Hamilton Ashworth


### *A WORD OF CAUTION TO WOOLEN MILL OPERATIVES.*

By Hamilton Ashworth.

Woolen mills are as a rule healthier places to work in than cotton or jute mills, because in the former the wool is kept carefully oiled throughout the various processes from the time it goes into the picker-house until it is spun into yarn or woven into fabric, when the oil is washed out; while cotton is worked without oil, on machinery that runs at a high rate of speed, so that the dust and fibre is much of it thrown into the air, where it floats continually, and getting into the eyes and nasal and bronchial passages of the operatives, causes irritation of these parts and often more serious inflammation.

Yet the very oil with which the fibres of wool are oiled in woolen mills may become a source of danger not less serious than the cotton flyings in cotton mills. It is still customary with owners of small mills located in the Western and less populous Eastern districts, to take common soap greese from the neighboring farmers and wool growers in exchange for goods or at a low cash price. This grease is sometimes very nice, sweet and pure, but more often it is such grease as the farmer's wives would not even make soap of, and is full of putrefaction and microscopic germs of disease. If this were all made into soap for scouring the cloth its unwholesomeness would be greatly diminished, if not destroyed altogether, for the strong lye acts as deodorizer and purifyer; but more often a large portion of this grease is used in its raw state for oiling the wool, and is thus brought directly and intimately into contact with the hands of the operatives.

It also penetrates into their clothing, and by the natural act of rubbing the face and scratching the head is distributed about the eyes and nose, and into the scalp, so that hardly a portion of the body escapes contact with this foul grease. This refuse grease always contains more or less fatty acid, productive of humors, and frequently the fat of animals that have died of disease. If this comes into contact with the blood through cuts or abrasions of the skin, it is likely to produce blood poisoning, with its distressing and frequently fatal results.

Sickness would occur more often if it were not for the fact that the hands of the operatives are generally more or less covered with machine oil, which is most always largely, if not wholly, a petroleum product, and is a skin curative and preventive of humours and poisoning.

The safest way, and the cleanest way, is to use only mineral oils on wool, or at least an oil which shall be composed principally of petroleum fat, and to make all the soap grease directly into soap. Mineral fats are now made with such care and skill that they are equal if not superior to the finest animal oils for oiling wool, and will lubricate and wash out perfectly. They are absolutely free from fatty acid or anything injurious to the skin. Indeed they are the bases of most of the ointments for healing skin diseases.

Much of the "breaking out" on the hands, arms and face, and much of the itching of the scalp, now so common among woolen mill operatives, might be found to cease with a change from animal to mineral oil.

Manufacturers will consider the health and comfort of their employees as well as their own health now that this matter has been brought to their attention.

